# Clinical patterns of primary stabbing headache: a single clinic-based prospective study

**DOI:** 10.1186/s10194-017-0749-7

**Published:** 2017-04-11

**Authors:** Dong Yeop Kim, Mi Ji Lee, Hyun Ah Choi, Hanna Choi, Chin-Sang Chung

**Affiliations:** 1 Department of Neurology, Samsung Medical Center, Sungkyunkwan University School of Medicine, Seoul, Korea; 2 Department of Neurology, Eulji University Hospital, Eulji University School of Medicine, Daejeon, Korea

**Keywords:** Primary stabbing headache, Clinical course, Pattern, Remission, Prognosis

## Abstract

**Background:**

The clinical features and disease courses of primary stabbing headache (PSH) are diverse. We aimed to identify distinct clinical patterns of PSH.

**Methods:**

We prospectively screened consecutive first-visit patients who presented with stabbing headache at the Samsung Medical Centre Headache Clinic from June 2015 to March 2016. Demographics, headache characteristics, and disease courses were prospectively evaluated. After discerning factors related to the chronicity at the time of presentation, clinical patterns were identified based on the frequency (daily vs. intermittent), clinical course (remitted or not), and total disease duration (<3 or >3 months).

**Results:**

In the 65 patients with PSH included in this study, monophasic (*n* = 31), intermittent (*n* = 17), and chronic daily (*n* = 12) patterns were identified. The median disease durations were 9 days for monophasic PSH, 9 months for chronic daily PSH, and 2 years for intermittent PSH. The features of monophasic PSH were greater severity, single and side-locked locations, more attacks per day, daily occurrence, and good treatment response. Chronic daily PSH was associated with female predominance, longer-lasting stabs, and multiple or migrating locations on bilateral or alternating sides. The characteristics of intermittent PSH included female predominance and sporadic stabs with less intensity.

**Conclusions:**

Our study demonstrated distinct clinical patterns of PSH. In addition to help early recognition of disease, our findings suggest different pathophysiologic mechanisms. Future prospective studies are required to reveal the etiologies of these different PSH patterns and their optimal treatment strategies.

## Background

Primary stabbing headache (PSH) is a primary headache disorder characterised by transient and localised pain attacks occurring as either a single stab or series of stabs [1]. Since this disorder was first described as “ophthalmodynia periodica” in 1964, the nomenclature was revised to PSH in the second edition of the International Classification of Headache Disorders (ICHD-2) [2]. The diagnostic criteria for PSH have recently been revised in the ICHD 3rd edition beta version (ICHD-3 beta), particularly regarding the location of the pain and associated symptoms [3]. Most PSH patients have very brief attacks [4–7]. Migraineurs often have PSH, during or independently from migraine attack [8]. PSH is one of indomethacin-responsive headaches. Currently, the pathophysiologic mechanisms of PSH are not fully understood. Although most PSH are idiopathic and benign, secondary causes have been reported [9, 10].

While PSH is common in population-based subjects, a diagnosis of PSH is relatively infrequent in clinic-based studies [4, 5, 11]. Less impact on daily life and self-limiting nature of PSH might be a reason for not visiting a clinic [8]. However, patients visiting a headache clinic may have different features. Variable disease durations and attack frequencies have been reported in different clinic-based cohorts with PSH [5, 12]. To date, the clinical patterns and related clinical outcomes have not been systematically examined in prospective studies. The identification of clinical patterns is useful for the diagnosis of primary headache disorders [13, 14]. Furthermore, clarifying the clinical characteristics of the different clinical courses can provide insight into the underlying pathophysiology of the disease. In the present study, we aimed to investigate the clinical patterns of PSH and related characteristics.

## Methods

### Patient selection

We prospectively screened consecutive first-visit patients who presented with stabbing headaches at the Samsung Medical Centre Headache Clinic from October 2015 to December 2016. Patients were screened even when their stabbing headache was not the main purpose of their visit. Of the patients who agreed to participate in this study, those who were diagnosed with ICHD-3 beta 4.7 PSH were included. Patients were excluded if any primary or secondary headache disorders other than PSH were diagnosed at the first or follow-up visits. When the patients reported the persistence of localised stabs in the occipital nerve territory, they were assessed for any tenderness or response to occipital nerve blocks in order to exclude occipital neuralgia. The Samsung Medical Center Institutional Review Board has approved this study.

### Evaluations

Before the screening, all patients completed standard questionnaires regarding their demographics, headache characteristics and medical and social histories. When patients were identified to have any headaches of stabbing nature, they were asked to participate this study. Two investigators (M.J.L. and H.A.C.) interviewed all of the patients with a structured questionnaire that was specifically designed for assessing stabbing headaches. The questionnaire included questions on the duration, frequency, onset, evolution, and previous history of stabs; accompanying PSH-specific symptoms, such as allodynia, jolts, and body jabs; and differentiating symptoms, such as skin rash. Premorbid migraine was self-screened with ID-migraine^TM^ and confirmed by the investigators [15]. Physician-diagnosed depression, anxiety, and panic disorders were self-reported by patients. Insomnia was also self-reported. Stress perceptions during the previous 3 months prior to visit were evaluated with numeric rating scales of 0 – 100, modified from Schramm et al. [16]. The patients were followed by the investigators with a follow-up questionnaire that evaluated their clinical outcomes and treatment responses.

The diagnoses of PSH were made based on the ICHD-3 beta criteria for 4.7 Primary Stabbing Headache [1]. Brain or cervical imaging studies were performed when clinically required to exclude secondary causes.

### Determination of disease course

Remission was defined as the complete resolution of headaches for more than 14 days for patients with daily PSH and more than twice the average interval of stabs in patients with non-daily PSH. If the patients’ symptoms already remitted before the first visit, the clinical course was considered remitted without follow-up. Clinical courses of patients with persistent symptoms were determined when followed up for >14 days. Follow-up was terminated when the patients showed remission. After excluding three patients with <14 days of follow-up, the median duration of follow-up was 60 days (range, 14 – 325). If the patient’s symptoms were too infrequent to detect remission (i.e., 2–3 attacks per year) during the follow-up period, the clinical courses were determined at the investigator’s discretion. The total duration of disease is presented as the number of days since symptom onset to the last attack regardless of treatment.

### Pattern identification

The occurrence pattern and disease course were used to identify the clinical patterns. In patients in remission and total disease durations less than 3 months, the clinical course was considered monophasic. The pattern could not be determined in unremitted patients with total disease durations less than 3 months. Patients with total disease durations over 3 months and daily stabs were characterised with a chronic daily pattern. In patients with a chronic daily pattern, a chronic progressive pattern was identified if the frequency, severity, or extent increased. An intermittent pattern was defined as the non-daily occurrence of stabs, without predefined frequency criteria. Patients with intermittent patterns were diagnosed with clustering or sporadic patterns based on the daily stab occurrence pattern. A clinical pattern was considered distinct if 10 or more patients had a similar course.

### Statistical analysis

Chi-square and Fisher’s exact tests were used to compare categorical variables among the groups, while Student’s *t*-tests and Mann–Whitney tests were performed for group comparisons of continuous variables according to their distribution. The statistical analyses were performed with SPSS (version 18.0; IBM Corporation, Armonk, NY, USA). Differences were considered significant at a two-tailed p value less than 0.05.

## Results

### Study subjects

Among the 71 patients screened during the study period, six were excluded because they had diagnoses other than PSH at the baseline or follow-up visit (occipital neuralgia, *n* = 1; trigeminal neuralgia, *n* = 2; postherpetic neuralgia, *n* = 1; active herpes zoster, *n* = 1; and spinal cord tumor, *n* = 1). Finally, a total of 65 patients with PSH were included in the analysis.

### Patient characteristics

The demographics and headache characteristics of the study subjects are listed in Table 1. Our study subjects showed a female preponderance (69.2%) and wide range of age (median, 54 years; range, 25–83). The symptoms occurred as a pattern of single (15.4%) or series (84.6%) of stabs. The majority of patients showed a daily occurrence (72.3%), but an intermittent pattern of occurrence on a monthly (12.4%) or yearly (13.8%) basis was also reported. A history of stabbing headache before the index event was reported for 14 (21.5%) patients.

**Table 1:** Patient characteristics

Age (y)	54 (25 – 83)
Female sex	45 (69.2%)
Disease duration prior to visit (d)	40 (1 – 13,140)
Follow-up (d)	44 (3 – 325)
Total disease duration (d)	40 (1 – 13,170)
Severity	7 (2 – 10)
Duration (s)	1 (<1 – 60)
Affected territory (not exclusive)	
Occipital nerve	55 (84.6%)
Trigeminal nerve	8 (12.3%)
Multifocal	16 (24.6%)
Side	
Side-locked	45 (69.2%)
Bilateral or alternating	20 (30.8%)
Migrating location	15 (23.1%)
Stab frequency per day	
1 (sporadic stab)	10 (15.4%)
2 – 10	26 (40%)
10 – 30	10 (15.4%)
30 – 100	11 (16.9%)
> 100	8 (12.3%)
Pattern of occurrence	
Daily	47 (72.3%)
Intermittent	
A few per month	9 (13.8%)
A few per year	9 (13.8%)
Premorbid migraine	27 (41.5%)
Previous history of stabbing headaches	14 (21.5%)
Allodynia	24 (36.9%)
Jolt	48 (73.8%)
Bodily jab	8 (12.3%)
Prognosis	
Remission	28 (43.1%)
Persistence	37 (56.9%)
Mode of complete remission	
Spontaneous	17 (45.9%)
By treatment	20 (54.1%)
Treatment	
Indomethacin	8 (12.3%)
Prednisolone	11 (16.9%)
Gabapentin	13 (20.0%)
Tricyclic antidepressants	6 (9.2%)

The data are presented as median (range) or number (%)

### Disease courses

**Fig. 1:**
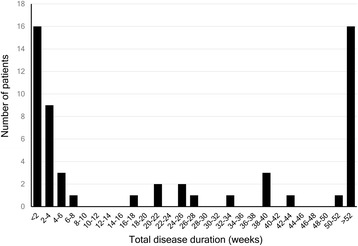
Distribution of the total disease durations

**Fig. 2:**
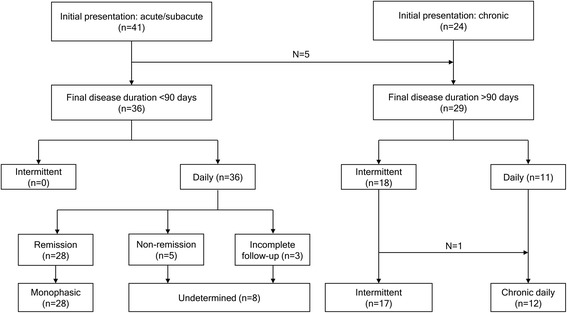
Algorithm of pattern identification

The total disease durations were widely distributed (median, 40 days; range, 1–13,170 days; Fig. 1). When the disease durations were stratified into 2-week strata, two peaks in the lowest and highest strata were observed (Fig. 2). Remission was achieved in 28 (43.1%) patients, while 37 (56.9%) patients showed persistent symptoms. The mode of remission was spontaneous in 17 (45.9%) patients and due to pharmacologic treatment in 20 (54.1%).

Demographics and headache characteristics of study subjects were compared according to the chronicity of the PSH at the time of presentation (Table 2). Patients with acute/subacute PSH (*n* = 41) had a more severe (*p* = 0.012) and side-locked (*p* = 0.044) stabs than patients with chronic PSH (56.0%, *p* = 0.068). Patients with chronic PSH (*n* = 24) had a female predominance (*p* = 0.015) and a lower frequency of attacks per day (*p* = 0.019). Most patients with acute/subacute PSH had daily stabs (92.5%), while patients with chronic PSH had a more intermittent pattern (p for trend < 0.001). Thirteen (31.7%) patients with acute PSH had a history of PSH (*p* = 0.009) compared to patients with chronic PSH. The prevalence of associated allodynia, jolts, and bodily jabs did not differ between the two groups (*p* = 0.672, >0.999, and 0.149, respectively). Other medical, psychiatric, and social comorbidities did not differ between the groups.

**Table 2:** Patient demographics and characteristics according to chronicity at the initial visit

	Acute/subacute (*n* = 41)	Chronic (*n* = 24)	*p*
Age (y)	54 (48–61)	55 (44–62)	0.822
Female sex	24 (58.5%)	21 (87.5%)	0.015
Severity (Numeric Rating Scale)	8 (7 – 9)	7 (5 – 8)	0.012
Duration (s)	1 (1 – 3)	1 (1 – 3)	0.348
Affected territory (not exclusive)			
Occipital	37 (90.2%)	18 (75.0%)	0.154
V1	4 (9.8%)	4 (16.7%)	0.454
Multiple	7 (17.1%)	9 (37.5%)	0.065
Side			0.044
Side-locked	32 (78.0%)	13 (54.2%)	
Bilateral or alternating	9 (22.0%)	11 (45.8%)	
Migrating location	7 (17.1%)	8 (33.3%)	0.133
Stab frequency per day			0.019*
1 (sporadic stab)	4 (9.8%)	6 (25.0%)	
2 – 10	15 (36.6%)	11 (45.8%)	
10 – 30	6 (14.6%)	4 (16.7%)	
30 – 100	9 (22.0%)	2 (8.3%)	
> 100	7 (17.1%)	1 (4.2%)	
Pattern of occurrence (either single or series of stabs)			<0.001*
Daily	39 (95.1%)	8 (33.3%)	
A few per month	2 (4.9%)	7 (29.2%)	
A few per year	0 (0.0%)	9 (37.5%)	
Disease duration prior to visit (d)	10 (7 – 30)	1,095 (195 – 2,920)	0.000
Follow-up (d)	34 (14 – 64)	49 (29 – 98)	0.086
Total disease duration (d)	15 (6 – 30)	987 (274 – 2,291)	0.000
Complete remission	33 (80.5%)	4 (16.7%)	<0.001
Mode of remission			0.109
Spontaneous	17 (51.5%)	0 (0.0%)	
By treatment	16 (48.5%)	4 (100.0%)	
Treatment response^a^			
Indomethacin	1/3 (33.3%)	4/5 (80.0%)	0.464
Prednisolone	6/7 (85.7%)	0/4 (0.0%)	0.015
Gabapentin	6/9 (66.7%)	1/4 (25.0%)	0.266
Tricyclic antidepressants	1/3 (33.3%)	0/3 (0.0%)	>0.999

The data are presented as median (interquartile range) or number (%)

*p for trend; ^a^Only in patients treated by medications (numbers shown in the table)

Regarding the clinical course, patients with acute/subacute PSH had a median disease duration of 15 (interquartile range, 6–31) days. They showed a high rate of remission (80.5%), which occurred either spontaneously (51.5%) or as a result of pharmacologic treatment (48.5%). All patients with remission had daily occurrence of stabs at baseline. Five patients with initial acute/subacute presentation progressed to chronic PSH (*n* = 3 with daily stabs, *n* = 2 with a few stabs per month; Fig. 2). All the five patients already had more than 2 months of disease duration at the first visit. Patients with chronic PSH had a low rate of remission (16.7%) and longer disease duration (median, 1,139 days; interquartile range, 275–1,950). Although the number of patients in each group was very small, the steroid response was more prominent in patients with acute/subacute PSH (*p* = 0.015). Patients with chronic PSH showed a high response rate (80.0%) to indomethacin.

### Clinical patterns of PSH

**Fig. 3:**
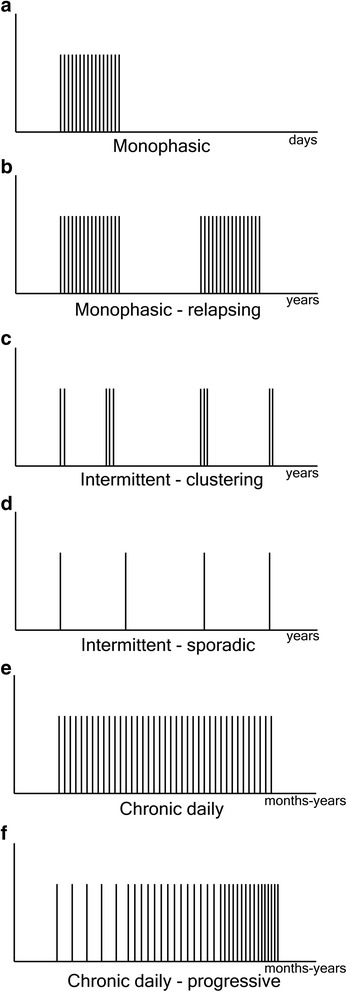
Clinical patterns of primary stabbing headache. **a**-**b** Monophasic pattern (*n* = 28). In the monophasic group, a relapse was reported in 10 patients (35.7%). **c**-**d** Intermittent pattern (*n* = 17). Stabs occur in cluster (**c**) or sporadically (**d**). **e**-**f** Chronic daily PSH (*n* = 12). Six patients reported a progressive course (**f**)

The algorithm of pattern identification is shown in Fig. 2. The typical patterns are illustrated in Fig. 3. Twenty-eight patients exhibited the monophasic pattern (Fig. 3a–b). In the monophasic group, 10 patients (35.7%) recalled that they had a similar episode of PSH which remitted before the index event (Fig. 3b; monophasic-relapsing). An intermittent pattern was observed in 17 patients (Fig. 3c–d). Among them, 10 patients showed a clustering of stabs (Fig. 3c), while 7 reported only sporadic stabs (Fig. 3d). Among the 12 patients with chronic daily PSH (Fig. 3e–f), a progressive pattern was identified in six patients (Fig. 3f).

The characteristics of the patients with different clinical courses are summarised in Table 3. The median disease durations were 13 days for monophasic PSH, 9 months for chronic daily PSH, and 2 years for intermittent PSH. The features related to monophasic PSH were greater severity (*p* = 0.033 vs. intermittent PSH), single and side-locked locations (*p* = 0.010 and 0.010, respectively; vs. chronic daily PSH), more frequent attacks per day (*p* = 0.001 vs. intermittent PSH), daily occurrence (*p* < 0.001 vs. intermittent PSH), and good response to treatment. Chronic daily PSH was associated with longer-lasting stabs (*p* = 0.071 vs. monophasic PSH and *p* = 0.064 vs intermittent PSH), and multiple or migrating locations (*p* = 0.010 and 0.017, respectively; vs. monophasic PSH) on bilateral or alternating sides (*p* = 0.010 vs. monophasic PSH). Although statistically not significant, allodynia and bodily jabs were most frequently reported in patients with chronic daily PSH, while jolts were observed less than the other patterns. The characteristics of intermittent PSH included female predominance and sporadic stabs with less intensities, while the other features were similar to or between the patients with monophasic and chronic daily PSH.

**Table 3:** Patient characteristics for different clinical courses

	Monophasic (M; n = 28)	Intermittent (I; n = 17)	Chronic daily (C; n = 12)	*P* ^*^		Post-hoc	
					M vs. C	M vs. I	C vs. I
Age (y)	54 (48–61)	57 (48–63)	56 (39–62)	0.970	0.871	0.879	0.825
Female sex	17 (60.7%)	15 (88.2%)	9 (75.0%)	0.203	0.484	0.088	0.622
Chronicity at presentation	0 (0.0%)	16 (94.1%)	8 (66.7%)	<0.001	<0.001	<0.001	0.130
Follow-up (d)	34 (20–63)	43 (22–81)	72 (51–112)	0.020	0.006	0.560	0.048
Total disease duration (d)	13 (5–22)	1,460 (574–3,684)	269(155–398)	<0.001	<0.001	<0.001	0.001
Severity (Numeric Rating Scale)	8 (6–10)	7 (5–8)	7 (5–9)	0.114	0.226	0.046	0.575
Duration (s)	1 (1–3)	1 (1–3)	3 (1–9)	0.163	0.128	0.629	0.064
Affected territory (not exclusive)							
Occipital	25 (89.3%)	14 (82.4%)	9 (75.0%)	0.262	0.341	0.658	0.669
V1	2 (7.1%)	2 (11.8%)	3 (25.0%)	0.205	0.149	0.626	0.622
Multiple	4 (14.3%)	4 (23.5%)	7 (58.3%)	0.008	0.008	0.452	0.119
Side				0.015	0.021	0.284	0.274
Side-locked	23 (82.1%)	11 (64.7%)	5 (41.7%)				
Bilateral or alternating	5 (17.9%)	6 (35.3%)	7 (58.3%)				
Migrating location	4 (14.3%)	4 (23.5%)	6 (50.0%)	0.031	0.041	0.452	0.236
Stab frequency per day				0.118	0.476	0.001	0.018^*^
1 (sporadic stab)	2 (7.1%)	7 (41.2%)	0 (0.0%)				
2–10	9 (32.1%)	7 (41.2%)	7 (58.3%)				
10–30	6 (21.4%)	2 (11.8%)	2 (16.7%)				
30–100	6 (21.4%)	1 (5.9%)	2 (16.7%)				
>100	5 (17.9%)	0 (0.0%)	1 (8.3%)				
Pattern of occurrence				0.060	0.300	<0.001	<0.001
Daily	28 (100.0%)	0 (0.0%)	11 (91.7%)				
A few per month	0 (0.0%)	8 (47.1%)	1 (8.3%)				
A few per year	0 (0.0%)	9 (52.9%)	0 (0.0%)				
Migraine history	14 (50.0%)	6 (35.3%)	4 (33.3%)	0.314	0.491	0.372	>0.999
Allodynia	10 (35.7%)	4 (23.5%)	6 (50.0%)	0.605	0.490	0.513	0.236
Jolts	22 (78.6%)	14 (82.4%)	6 (50.0%)	0.131	0.130	>0.999	0.106
Bodily jabs	4 (14.3%)	1 (5.9%)	3 (25.0%)	0.636	0.410	0.635	0.279
Depression	4 (14.3%)	0 (0.0%)	0 (0.0%)	0.101	0.297	0.281	>0.999
Anxiety	3 (10.7%)	0 (0.0%)	0 (0.0%)	0.158	0.541	0.279	>0.999
Panic disorder	1 (3.6%)	0 (0.0%)	0 (0.0%)	0.702	>0.999	>0.999	>0.999
Caffeine consumption	22 (78.6%)	13 (76.5%)	8 (66.7%)	0.564	0.451	>0.999	0.683
Smoking	4 (14.3%)	1 (5.9%)	3 (25.0%)	0.636	0.410	0.635	0.279
Insomnia	7 (25.0%)	5 (29.4%)	5 (41.7%)	0.365	0.453	0.743	0.694
Stress scale	60 (30–71)	50 (30–60)	65 (35–80)	0.358	0.412	0.382	0.161

The data are presented as median (interquartile range) or number (%)

^*^Kruskal-Wallis tests were performed on continuous variables, and linear-by-linear association tests were performed on categorical variables

## Discussion

In this prospective study, we focused on the patterns and clinical courses of PSH. The key findings were the following: 1) PSH presented with either an acute or chronic course, 2) patients with acute/subacute and chronic PSH had different demographics and headache characteristics, thus implicating different pathophysiologies, and 3) clinical patterns of PSH are either monophasic, intermittent, or chronic daily patterns.

Previous descriptive studies of PSH have reported that both population-based and clinic-based subjects showed a wide range of disease durations, which ranged from days to several years [4, 5]. The results of our study were in line with the results of the previous studies, showing diverse disease durations that ranged from 1 day to 18 years. We found that the distribution of the disease durations had two peaks (<2 weeks and > 1 year), which suggested the presence of two distinctive clinical courses: acute/subacute and chronic. To date, chronic PSH has not been classified as an independent entity in the ICHD-3 beta. However, our study results showed that chronic PSH was not uncommon.

Previous studies have reported different demographics and characteristics [4, 7, 12, 17, 18]. Our results showed that different characteristics were involved in the chronicity. The characteristics of patients with acute/subacute PSH included more localised locations, greater intensity, more stabs per day, and daily occurrence. Moreover, a history of PSH was more common in the patients with acute/subacute PSH, which suggested a high relapse rate in these patients. In contrast, chronic PSH was associated with female predominance, multifocal and bilateral locations, and both daily and intermittent patterns of occurrence.

Based on these findings, distinct clinical patterns were identified in PSH. The most common pattern was monophasic, which was characterised by the daily occurrence of a series of stabs that lasted days to weeks and a high rate of complete remission. In contrast, the intermittent pattern was defined as a few attacks of sporadic or clustering stabs over a month or year. The stab occurrences did not exceed one day. In this subgroup, pharmacologic treatment was not routinely performed because of its low frequency and negligible impact on routine life. Finally, chronic daily PSH was another extreme of this disorder. Although the frequencies of stabs per day varied among the patients, they had daily or near daily attacks. Some patients reported an evolution from intermittent to daily patterns, which suggested disease progression.

Different disease patterns may have pathophysiological implications, such as different origins of pain or mechanisms of chronification. The monophasic disease might be explained by acute irritation of the peripheral branches of the trigeminal or occipital nerves [5, 19], which resembles a zoster sine herpete that presents with acute-onset neuralgia in the absence of an antecedent rash [20, 21]. Due to assumptions of an inflammatory etiology, we tried steroid treatments for the patients suffering from high-frequency and severe-intensity stabs, and this treatment resulted in an excellent response. Based on these uncontrolled treatment experiences of selected patients, the steroid treatments had the most immediate effects compared with the other therapeutic agents. These results suggest that further investigations of a viral etiology are worthwhile for revealing the mechanisms and optimal treatments of monophasic PSH. The chronic daily pattern was associated with multiple or migrating locations, slightly longer-lasting stabs, frequent allodynia, and bodily jabs, which suggested central sensitisation of the second-order nociceptive neurons [22, 23]. The mechanism of central sensitisation in PSH might be different from that of migraine because the presence of migraine or factors related to migraine chronification were not associated with chronic daily PSH. It remains to be investigated whether the early treatment of monophasic or intermittent PSHs can reduce the development of chronic daily PSH. The intermittent pattern had features that were similar to or in between monophasic and chronic daily PSH. Future studies need to investigate whether intermittent PSH is an independent entity or a milder form of monophasic or chronic daily PSH.

Although a controversy exist, brain imaging is recommended by some experts to exclude secondary causes of PSH [24]. Investigation of underlying secondary causes may be more important in emergency rooms and tertiary headache clinics [25–27]. Pattern recognition might be helpful for identifying PSHs as a primary headache disorder. In our data, unremitted daily PSHs of recent onset is not common in patients with PSH, warranting an appropriate evaluation for secondary causes. Prevalence of secondary causes in each clinical pattern should be investigated in future studies.

This study has several strengths. This is the first study to focus on the clinical course of PSH. The prospective study setting and homogenous disease-specific evaluation are the main strengths of this study. However, some limitations are also present. The subjects in this study might not represent the general population because they were sampled from a specialised headache clinic in a university hospital. Thus, the prevalence of the acute or chronic PSH cannot be generalized. In order to reduce the selection bias, we recruited patients with a broad spectrum of disease by screening patients whose main complaints were not a stabbing headache. Another limitation is that the treatments were not controlled in this observational study. Therefore, the response rates among drugs could not be directly compared. Finally, headache frequencies were not defined by prospective headache diary.

In conclusion, distinct clinical patterns, such as monophasic, intermittent, and chronic daily patterns, are associated with different clinical characteristics. Different pathophysiologic mechanisms may be present, thus warranting future prospective studies to reveal the etiologies of the different PSH patterns and optimal treatment strategies.
